# Cyclical aggregation extends in vitro expansion potential of human mesenchymal stem cells

**DOI:** 10.1038/s41598-020-77288-4

**Published:** 2020-11-24

**Authors:** Brent M. Bijonowski, Xuegang Yuan, Richard Jeske, Yan Li, Samuel C. Grant

**Affiliations:** 1 Department of Chemical and Biomedical Engineering, FAMU-FSU College of Engineering, Florida State University, 2525 Pottsdamer St., Tallahassee, FL 32310 USA; 2 The National High Magnetic Field Laboratory, Florida State University, Tallahassee, FL USA; 3 Present Address: University of Münster, Münster, Germany

**Keywords:** Biotechnology, Stem cells

## Abstract

Mesenchymal stem cell (MSC)-based therapy has shown great promises in various animal disease models. However, this therapeutic potency has not been well claimed when applied to human clinical trials. This is due to both the availability of MSCs at the time of administration and lack of viable expansion strategies. MSCs are very susceptible to in vitro culture environment and tend to adapt the microenvironment which could lead to cellular senescence and aging. Therefore, extended in vitro expansion induces loss of MSC functionality and its clinical relevance. To combat this effect, this work assessed a novel cyclical aggregation as a means of expanding MSCs to maintain stem cell functionality. The cyclical aggregation consists of an aggregation phase and an expansion phase by replating the dissociated MSC aggregates onto planar tissue culture surfaces. The results indicate that cyclical aggregation maintains proliferative capability, stem cell proteins, and clonogenicity, and prevents the acquisition of senescence. To determine why aggregation was responsible for this phenomenon, the integrated stress response pathway was probed with salubrial and GSK-2606414. Treatment with salubrial had no significant effect, while GSK-2606414 mitigated the effects of aggregation leading to in vitro aging. This method holds the potential to increase the clinical relevance of MSC therapeutic effects from small model systems (such as rats and mice) to humans, and may open the potential of patient-derived MSCs for treatment thereby removing the need for immunosuppression.

## Introduction

The therapeutic potential of human mesenchymal stem cells (MSCs) has been revealed in numerous studies due to their immunomodulatory effects^1–4^, enhancement of tissue regeneration and angiogenesis in ischemic injuries^5,6^, and as a potential source of therapeutic exosomes^7–9^. Yet the in vivo therapeutic potency has been done predominately in animal models which require orders of magnitude fewer cells compared to human subjects. Therefore, MSCs have not been widely applied to clinical trials as expected. One of the major limitations for clinical applications of MSCs is imposed by in vitro scale-up of deliverable cells^10^. Additionally, there is a supply disconnect because cells for rodent models can be cultured directly before injection, while clinical studies rely on defrosting ampules of cells and injecting them without proper cellular relaxation^11^. The majority of in vitro and in vivo studies utilize bone marrow or adipose tissue-derived MSCs due to their relative ease of extraction and density of viable cells^12^. Yet there are still too few cells recovered from any one donor. Thus, donor pooling is utilized in order to achieve adequate dosing in certain disease model such as graft-versus-host disease^13^. Ideally, patients would be treated with their own MSCs expanded at a large scale in vitro; however, this is limited because MSCs lose functionality as a result of extended in vitro culture expansion.

MSCs are very sensitive to their microenvironment and could adapt cellular events based on certain physiological cues such as oxygen or biophysical cues such as culture surface stiffness^14^. The culture adaption of MSCs can be beneficial under certain conditions such as hypoxia treatment and 3D aggregation culture, which are widely applied to preconditioning of stem cells^15^. In other cases, culture adaption to nutrient/oxygen enrichment and in vitro culture leads to cellular senescence or in vitro aging^11^. The long-term cultured MSCs may exhibit increased senescence, loss of differentiation potential, loss of beneficial secretory profile, and loss of proteostasis, which all contribute to a general loss of stem cell phenotype^11^. Proteostasis is essential to stem cell function and fate decisions^16^. It has been observed that as embryonic stem cells undergo in vitro aging, they gradually lose control over their proteome which further triggers cell death^16^. This is a critical control as accumulation of defects in daughter cells would be detrimental to the organism^17^. This defect accumulation is postulated as a direct result of a breakdown in the integrated stress response (ISR), which is a complex pathway that recognizes misfolded proteins and initiates a lockdown on protein synthesis, increases folding chaperones, and if proteostasis cannot be recovered, provides signals for autophagy^17,18^.

However, these effects can be mitigated through the MSC’s 3D aggregation process in which cells are integrated into a new cellular environment with a lower modulus similar to human tissue^19–23^. Additionally, aggregation promotes cell–cell interactions, which create new cell signaling events from either paracrine or biomechanical stimulation^24–26^. Both mechanisms may contribute to how aggregated MSCs have been shown to enhance multiple cellular functions, such as anti-inflammatory and regenerative potential^27,28^, stem cell genes^29,30^, and the increased ISR response^31^. However, cells within the aggregate have reduced proliferative potential, meaning that aggregation alone cannot solve the scale-up problem^30,32^.

For this reason, the hypothesis that cyclical aggregation of MSCs can extend the ability to counter in vitro culture stress and maintain functionality during in vitro culture expansion compared to planar culture. To accomplish this, early passage human adipose tissue-derived mesenchymal stem cells (hASCs) were either cultured on planar surfaces or cycled between aggregation and planar culture (i.e., cyclic aggregation-passaging or CP) for an equal passage number of planar expansions. Proliferation and population doubling times were recorded along with stem cell functions such as differentiation into adipogenic and osteogenic cells, expression of stem cell genes, and the level of senescence. It was observed that cyclical aggregation resulted in well-maintained proliferative capability, cellular doubling time, MSC morphology, mRNA levels of stem cell genes, the abilities of differentiation into adipocytes and osteoblasts, and reduced β-galactosidase activity over the 12 aggregation-planar expansions. Inhibition of the ISR pathway with GSK-2606414 reversed these effects and hASCs in vitro aged more rapidly. These results illustrate a novel culture method/system by which MSCs can be in vitro expanded while also maintaining functionality in long-term culture; thereby, opening up multiple clinical avenues for treatments that would be otherwise closed due to immunogenicity or functional decline due to MSC senescence.

## Results

### Aggregation of hASCs maintains naïve morphology and growth kinetics

**Figure 1:**
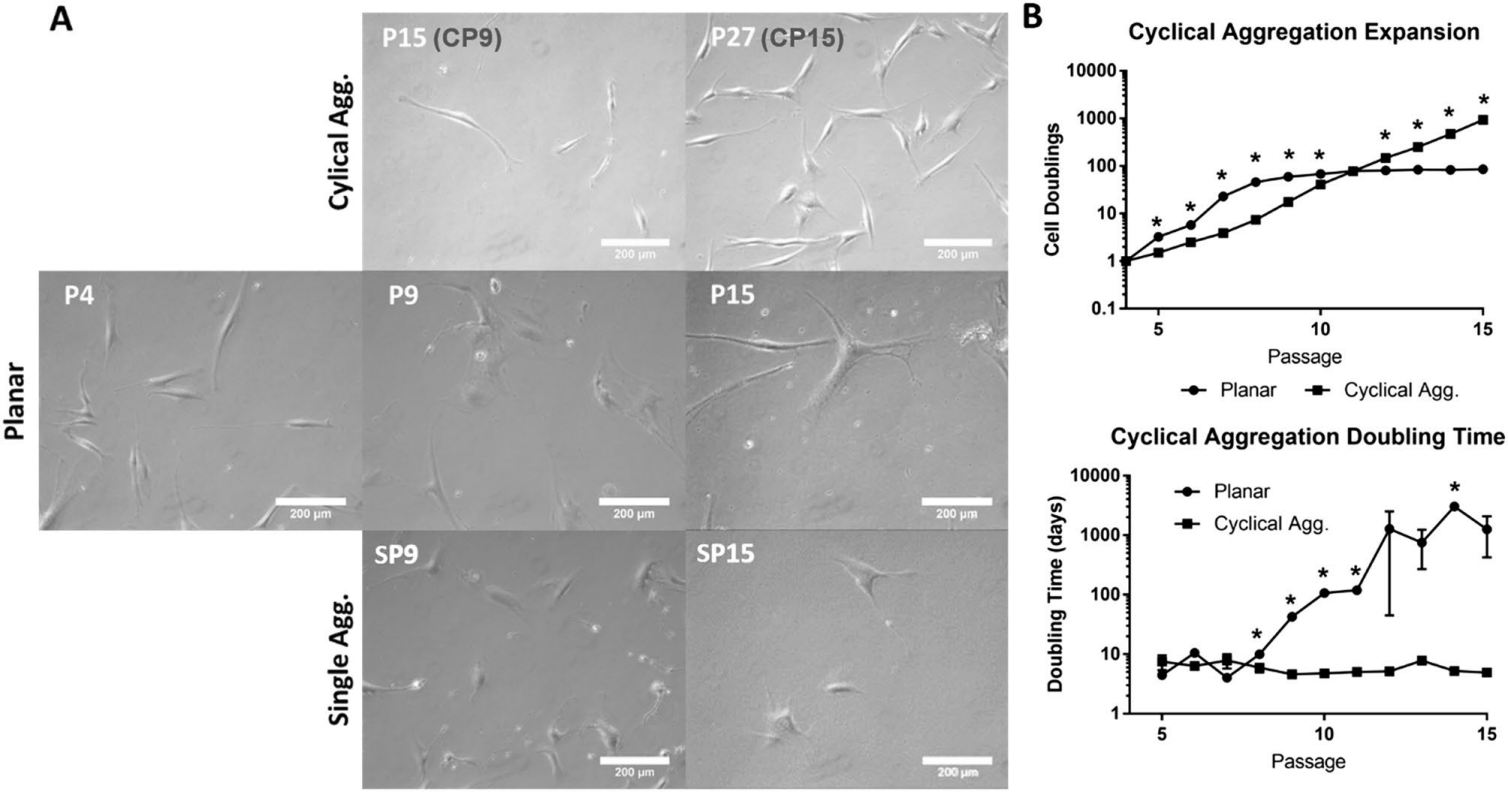
Assessment of ASC morphology along with growth kinetics to determine the effects of cyclical aggregation. (**A**) Morphologic assessment revealed the loss of spindle shape in planar expansion, yet morphology was maintained via cyclical aggregation. (**B**) Analysis of ASC doublings and doubling time revealed that while planar cells were able to expand readily during early passages, the cells were eventually outpaced by aggregated cells which also maintained doubling time. Data represents the mean of at least three independent determinations; errors represent the standard error in the mean. *p < 0.05.

To assess the effect of aggregation in maintaining cellular properties, the cellular morphology was monitored. Microscopic images revealed that as passage number increased via planar expansion, hASCs gradually lost their characteristic spindle shape (Fig. 1A). Single passage aggregation from passage 8–9 (SP9) and 14–15 (SP15) was able to partially recover the morphology. Cells from cyclical aggregation maintained the spindle shape at passage 15 (CP9) and 27 (CP15) (Fig. 1A and Suppl. Figure S1). Next, cellular proliferation and doubling time were investigated (Fig. 1B). Planar expansion led to an early growth pattern, but eventually cells stopped doubling, showing a stagnant population curve. Cyclical aggregation resulted in a continuous growth pattern over the 12 cycles of expansions, showing significantly more cells and a reduced doubling time than planar expansion.

### Integrated stress response affects aggregation-based ASC maintenance

**Figure 2:**
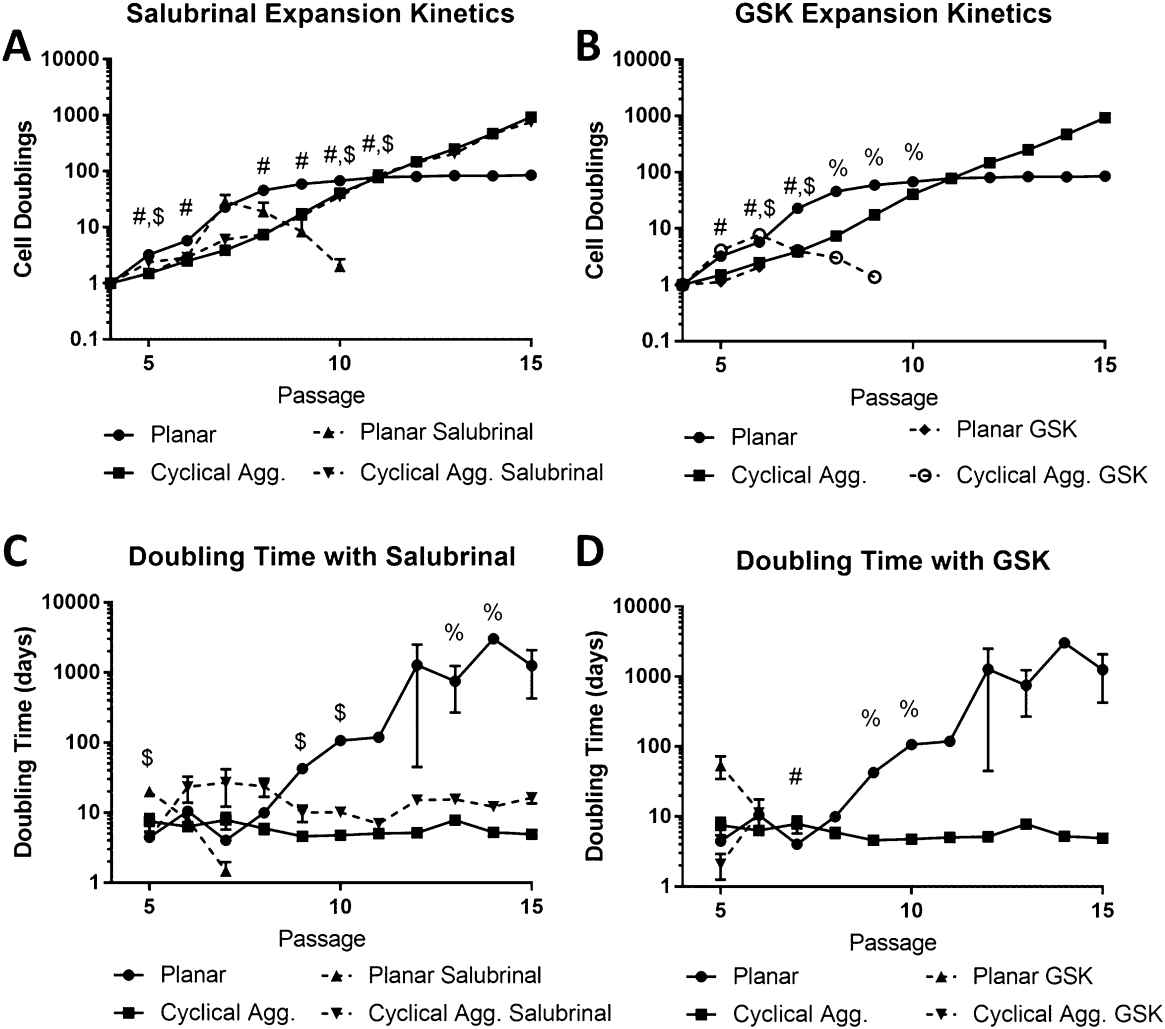
Integrated stress response (ISR) modulation shows that aggregation-induced signaling is responsible for maintenance of stem cell growth kinetics. Cell doublings under ISR response following treatments with (**A**) salubrinal (5 µM) and (**B**) GSK-2606414 (10 µM), which illustrate that inhibiting ISR results in accelerated cell death. Doubling time of ISR-modulated ASCs: (**C**) salubrinal (5 µM) and (**D**) GSK-2606414 (10 µM), revealing the ISR effect of cyclical aggregations on stem cell growth. Data represents the mean of at least three independent determinations; errors represent the standard error in the mean. # indicates *p* < 0.05 (planar vs. treatment), % indicates *p* < 0.05 (aggregation vs. treatment), $ indicates *p* < 0.05 (treated planar vs. treated aggregation).

Since ISR response is essential to maintaining stem cell function, and our study recently showed that aggregation of MSCs results in a heightened basal ISR response (manuscript submitted), the doubling time was assessed under ISR modulation with salubrinal, a growth arrest and DNA damage-inducible protein (GADD34) inhibitor which prevents ISR elevation, and GSK-2606414 (GSK), a phospho-protein kinase RNA-like endoplasmic reticulum kinase (PERK) inhibitor which prevents the cells from entering ISR. Treatment with salubrinal had no significant effect on doubling time of aggregates. However, planar cells eventually die after passage 10, showing a significant difference between the treated and untreated groups (Fig. 2A). Treatment with GSK affected both planar and aggregate cultures, and both conditions showed early cell death (Fig. 2B). Doubling times showed similar trend, i.e., aggregate cells treated with salubrinal doubled significantly slower than the untreated condition (Fig. 2C). GSK-treated cells had increased doubling times and the cell numbers dropped immediately, showing a negative doubling time (Fig. 2D). Additionally, planar cells treated with salubrinal exhibited better maintenance of spindle shape morphology up to passage 9 (Suppl. Figure S2), while GSK treatment resulted in accelerated loss of morphology (data not shown). Aggregated cells treated with salubrinal did not show significant change in morphology until CP9 (Suppl. Figure S2).

### Cyclical aggregation to maintains stemness and decreases senescence

**Figure 3:**
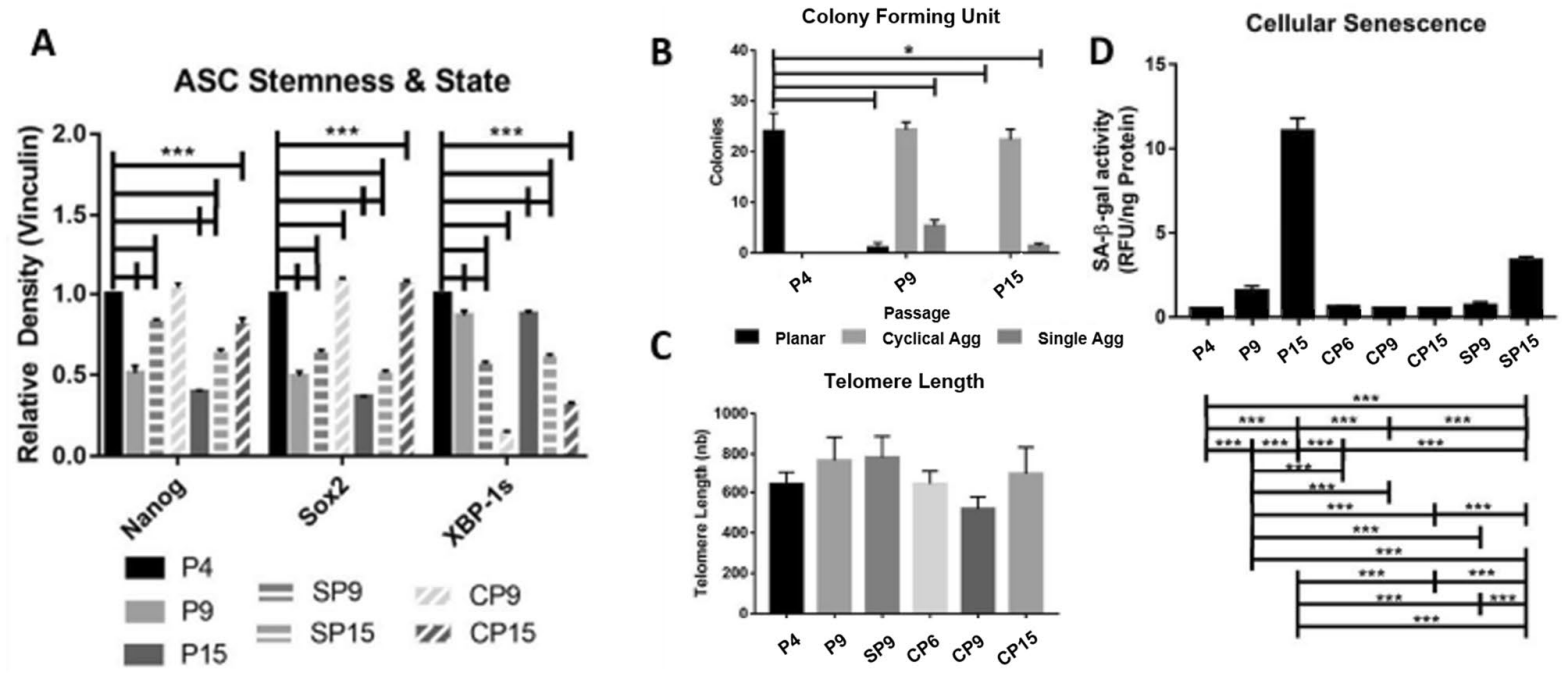
Assessment of stem cell functionality revealed signs of in vitro aging could be countered with aggregation treatment. (**A**) Quantification of Western blot analysis of stem cell markers Nanog and Sox-2 reveals that planar expansion leads to their loss, and an accumulation of junk protein XBP-1s. Cyclical aggregation increased Nanog and Sox-2 but decreased XBP-1s. (**B**) Colony-forming unit (CFU) assay was utilized to assess stem cell content in total cells, which reveals that planar cells loss this functionality; however, aggregation can retain stem cell content. (**C**) Telomere length was used to measure telomerase activity, a key component of stem cell rejuvenation. (**D**) β-galactosidase activity was used to measure the level of senescence, and a significant increase in activity was observed as passage number increased in planar culture. This could be prevented through cyclical aggregation. Data represents the mean of at least three independent determinations; errors represent the standard error in the mean. *p < 0.05, ***p < 0.001.

The cells from each time point were analyzed by Western blot. The results revealed that for ASCs under planar expansion, the expression of stem cell markers Nanog and Sox2 significantly decreased with passage number increase (i.e., P4, P9, and P15), and theexpression of the spliced misfolded protein signal XBP-1s significantly increased. Single aggregations resulted in a reversal of these trends at P9 and P15. Similarly, cyclically aggregated cells (P9 and P15) demonstrated the increased expression of Nanog and Sox2, and the decreased expression of XBP-1s (Fig. 3A). Next clonogenicity was measured and there was a significant drop in CFU number following planar expansion (P4, P9 and P15) (Fig. 3B and Suppl. Figure S3). Single aggregations did add some clonogenicity but was still significantly reduced compared to P4 ASCs. Cyclical aggregation, however, did not result in a significant change and maintained clonogenicity (Fig. 3B).

Telomere length was used to determine how passaging affects stem cells’ ability to replicate indefinitely. No significant trend was revealed for P4 and P9 cells of planar culture, single aggregated SP9 cells, and cyclical aggregated CP5, CP9, and CP15 cells (Fig. 3C). Cellular senescence was measured via senescence-associated β-galactosidase activity (β-gal), which showed a significantly increased activity from 0.521 ± 0.006 relative fluorescence units (RFU)/ng protein in planar P4 cells to 1.556 ± 0.270 RFU/ng protein and 11.040 ± 0.712 RFU/ng protein by passage 9 and 15 respectively. Additionally, single aggregated cells at SP9 exhibited the significantly reduced β-gal activity (0.706 ± 0.181 RFU/ng protein) compared to P9 planar cells, but not to P4 planar cells. Single aggregated cells at SP15 also showed the significantly decreased senescence (3.372 ± 0.191 RFU/ng protein) over the control group (P15 planar cells), yet was still significantly higher than P4 cells. Cyclical aggregation treatment did not significantly affect β-gal activity for cells at either CP6 (0.620 ± 0.062 RFU/ng protein), CP9 (0. 529 ± 0.014 RFU/ng protein), or CP15 (0.52 ± 0.001 RFU/ng protein) (Fig. 3D).

### Cyclical aggregation retains differentiation potential

A key component of stem cell function is differentiation potential. Adipogenic differentiation was characterized by staining differentiated cells with Oil Red O. Naïve cells showed strong positive staining of encapsulated oil red deposits; however, planar expansion to P9 and P15 resulted in a significant decline in number of oil red spots. Single aggregations had no effect of differentiation potential. Cyclical aggregation showed a similar level of Oil Red O staining to naïve cells (Suppl. Figure S4). Additionally, osteogenic differentiation was performed. ASCs at passage 9 and 15 showed low survival, and decreased differentiation, which was slightly improved with single aggregation. Cyclical aggregation, however, was able to maintain cellular viability and differentiation potential (Suppl. Figure S5).

### Rocker-based aggregation for potential process scale up

**Figure 4:**
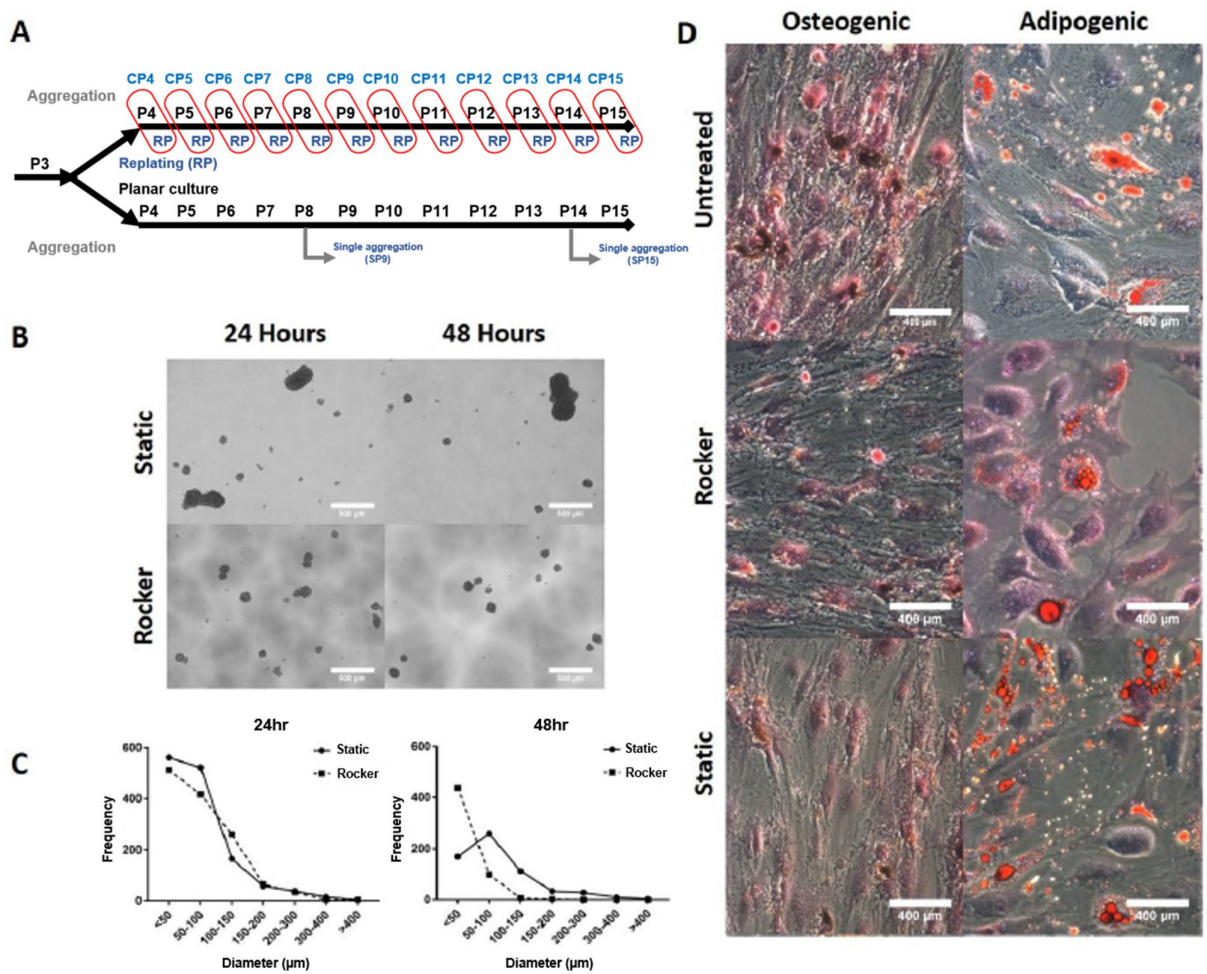
Analysis of static verses dynamic (rocker) aggregate formation in a low-attachment plate. (**A**) Experimental flow: For cyclical aggregation, hASCs were aggregated for two days before being trypsinized and replated until they reach confluence. For planar culture and single aggregation experiment, hASCs were cultured on tissue culture treated surfaces. (**B**) Microscopic images of aggregates formed under static and rocker-based aggregation culture at 24 and 48 h post-seeding. (**C**) Histogram of aggregate diameters for 24 and 48 h for both static and rocker-based aggregation. (**D**) Osteogenic and adipogenic differentiation potential following aggregation under static culture or rocker-based aggregation culture compared with untreated (non-aggregated) cells.

An alternative aggregation culture can be achieved by wave-motion to generate hASC aggregates as a potential scale-up process in a manner similar to cyclical aggregation (Fig. 4A). Preliminary experiments of aggregation culture of P4 hASCs showed that rocker-based aggregation results in a narrower aggregate diameter profile than that observed under static aggregation on a low-attachment surface (Fig. 4B,C). This is advantageous as it results in a homogenous population, thus minimizing differential influences of biomechanical stress induced by aggregates of different sizes. Similarly, whether aggregation method would alter differentiation potential was assessed, and no significant change was observed for ASCs using different aggregation methods (Fig. 4D).

## Discussion

MSCs pose significant therapeutic benefit; however, they must be expanded to reach the required numbers prior to being used in clinical trials. During in vitro expansion, MSCs rapidly adapt to the nutrient-enriched environment, experience in vitro aging, and eventually become senescent^33,34^. This work sets out to test whether cyclical aggregation, which has been shown to heighten MSC’s beneficial effects^35–37^ and restore naïve cellular function^38,39^, can preserve MSC properties under long term expansion. Cyclical aggregation-planar expansion is necessary because MSCs lose function during planar expansion but are functionally enhanced during aggregation culture. Yet aggregated cells proliferate slower than planer cells^40–42^. Additionally, improvements of cellular properties have been shown to last one passage beyond aggregation^42^. By coupling planar expansion with aggregation, cells can be expanded while minimizing accumulated stresses and retaining a more naïve phenotype. This method can be potentially used to scale up MSC aggregates in batches via Wave bioreactors^40^.

### Aggregation of ASCs prevents the loss of morphology and maintains cellular kinetics

Most studies utilize early passages of MSCs and ASCs up to passage 6 for experiments, due to loss of function during in vitro expansion^11^. A key hallmark of MSC aging is the loss of stem cell morphology, which was assessed at critical time points before and after the passaging. Images revealed that ASCs lost their characteristic spindle shape during planar expansion. This is in accordance with what has been observed by others^32,33^. For example, Yang et al. observed that passaging MSCs resulted in loss of cell shape and function as early as passage 5^33^. Previously, it has been shown that aggregation of ASCs results in the retention of stem cell morphology^41^. This study demonstrates that the cyclical aggregation likewise maintained the spindle morphology.

Beyond morphology, it was observed that the planar-cultured ASCs eventually become stagnant in growth kinetics, resulting in a significant reduction in expansion potential compared to cyclical aggregation. The process of aggregation did result in approximately 40% cell loss with each cycle (data not shown), which may be attributed to lack of proliferation in aggregates, increased caspase activity, and trypsinization^42,43^. However, ultimately the fact that aggregation maintains ASC proliferation and doubling time outweighs this loss, resulting in overall enhanced proliferation. Bartosh et al. also examined proliferation of MSCs following aggregation compared to planar culture, and observed an initial loss of proliferation ability followed by a spike in proliferation before losing the expansion potential and then returning to planar culture^39^.

### Modulation of ISR reverses aggregation-induced effects

Proteome control is essential to stem cell function^16^, and aggregation has been shown to rejuvenate ISR response^37^ and reestablish cellular function^17,28,44^. This study also explored if ISR is responsible for the effects observed following cyclical aggregation. As expected, salubrinal had no effect on aggregate culture, which may be attributed to the fact that aggregation already activates ISR, so further elevation of ISR does not affect the aggregated cells. However, there was a significant difference for planar culture with salubrinal-treated and untreated groups. For the first four passages, salubrinal-treated planar cells had similar cell doublings compared to the untreated group. However, after the initial expansion salubrial-treated cells died off. This can be explained by the fact that ISR prevents protein synthesis. GSK-2606414 prevents PERK signaling and was found to reverse aggregation-induced effects in proliferation. Therefore, under GSK-2606414 treatment, aggregate culture becoming similar to planar-expanded cells. Likewise, planar culture with GSK2606414-treated group accelerated cellular aging effects and led to cell senescence.

### Aggregation maintains stem cell markers, clonogenicity, and mitigates senescence

As stem cells age, they gradually lose the expression of stem cell markers such as Nanog and Sox-2. Kapetanou et al. recently assessed how Oct-4, Nanog, and Sox-2 shifted in MSCs from early passage, through middle passage, and into late passage. The authors revealed that all three proteins significantly decreased to at least 50% from early to late passage^45^. This is similar to what has been observed in this study for passages 9 and 15 cells respectively. Aggregation has been shown to significantly increase Oct-4, Nanog, and Sox-2 by at least two-fold^28,39^. This level of upregulation was not observed in this study, possibly due to the cells being expanded in planar culture for at least one passage following aggregation. The significant observation in this study is that stem cell markers did not decrease over time, but rather are maintained using cyclical aggregation method.

A recent publication by Bertolo et al. assessed how CFU and β-gal activity of MSCs fluctuated following 11 passages, and concluded that CFU decreased, while β-gal activity increased^4^. This is similar to the results observed herein. Aggregation has also been shown previously to increase CFU number, similar to the effects shown here^39^. Senescence has been shown to be decreased by approximately sevenfold following aggregation^28^. In current study, β-gal activity was also significantly reduced following aggregation of P9 and P15 hASCs. Additionally, the lack of a significant difference between any cyclical passage and the naïve P4 in stem cell markers, CFU activity, or β-gal activity confirms the hypothesis that cyclical aggregation can maintain stem cell characteristics.

### Cyclical aggregation maintains differentiation potential

Differentiation potential of ASCs was measured for adipogenic and osteogenic lineages in this study. Adipogenic differentiation ability was quickly lost by P9 and further degraded by P15. These effects could not be ameliorated by aggregation, showing that while aggregation can improve many functions, it has limitations in dedifferentiation potential. Cyclical aggregation on the other had maintained adipogenic differentiation potential. Similarly, osteogenic differentiation was strong initially, which was maintained by cyclical differentiation. However, planar expansion resulted in decreased cell viability, loss of cellular morphology, and decreased staining of calcium deposits. A similar reduction in adipogenic and osteogenic differentiation potential following planar expansion was observed by Yang et al.^33^. For adipogenic differentiation, a significant number of oil droplets at P4 was observed, but the number decreased significantly for P8 cells. This was corroborated by a drop in lipoprotein lipase and peroxisome proliferator-activated receptor gamma (PPARγ) which are both classical indicators of adipocytes. For osteogenic differentiation, a decrease in cellular viability and a reduction in calcium deposits in P8 cells were observed compared to P4. This was corroborated by a reduction in alkaline phosphatase expression.

## Conclusions

The therapeutic potentials of MSCs are undercut by the limitations found in large scale culture expansion for human clinical trials. This study utilized a novel culture strategy by cyclical aggregation and replating MSCs to address this engineering problem. MSCs that undergo this aggregation cycle preserved their stem cell phenotype compared to planar culture. This study also indicated that by perturbing the ISR induced by aggregation, MSCs can be expanded without jeopardizing their functionality. Specifically, cyclical aggregation can maintain cell morphology, growth kinetics, expression of stem cell proteins, clonogenicity, and differentiation ability and thus acts as an anti-senescence strategy during in vitro expansion. While more work is needed to further optimize the cycle of aggregation and planar expansion, this study demonstrates an important culture strategy as it lays the groundwork for creating a new biomanufacturing option for MSCs.

## Methods

### Culture of hASCs

Frozen hASCs at passage 1, in liquid nitrogen, were obtained from the Tulane Center for Stem Cell Research and Regenerative Medicine (hASCs were isolated from the liposate of de-identified healthy donors at Tulane). Cells from three donors (younger than 60 years of age, and the donors were not directly involved in this study) were used to carry out this study to ensure generalizability and significance of effects. hASCs were expanded with minimum essential medium-alpha (αMEM) (Life Technologies, Carlsbad, CA) supplemented with 1% penicillin/streptomycin (Life Technologies) and 10% fetal bovine serum (FBS; Atlanta Biologicals, Lawrenceville, GA) to form complete culture media (CCM) on 150-mm tissue culture Petri (TCP) dishes (Corning, Corning, NY) to a density of approximately 2500 cells/cm^2^ in a standard 5% CO_2_ incubator. The culture media were changed every 3 days. Cells from passage 4 (P4) were used as initial starting point for all experiments. All culture reagents were purchased from Sigma Aldrich (St. Louis, MO) unless otherwise noted.

### Aggregate formation

Once cells reached 90% confluence, the cells were trypsinized and pelleted. The pellet was resuspended in CCM at 1 × 10^5^ cells/mL. The cell suspension was pipetted at 1 mL per well in an ultra-low attachment (ULA) 6-well plate, for a total of 6 × 10^5^ cells. The aggregates were homogenized over 48 h. Multiple aggregates were observed per well. The morphologies and size-distribution of aggregates (100–400 µm in diameter) can be tracked and imaged with an Olympus IX70 microscope (Center Valley, PA) and were reported in our previous publication^40^. For rocker-based aggregation, The ULA plates were placed on a rocking platform (VWR International, Radnor, PA) in a standard humidified incubator (37 °C, 5% CO_2_) under controlled rocking angle of 8° and rocking speeds (i.e., 20 rpm) for 48 h. The rocking conditions generate wave motion of media under shear stress that supports spontaneous aggregation of hMSCs. Cells that went through one aggregation culture was defined as single-aggregation passage (SP).

### Cyclical aggregation and replating culture

After 48 h, aggregates were collected from each well, pelleted by centrifuge and washed with phosphate buffer saline (PBS). Then the hASC aggregates were incubated with 0.25% trypsin/ethylenediaminetetraacetic acid (EDTA) (Invitrogen, Grand Island, NY) for 10 min. Gentle pipetting was carried out every 5 min. hASC aggregates were digested into single cell suspension and then pelleted. After centrifugation, cell pellets were resuspended in CCM and counted. Small clusters existed in the suspension and were repleted together with single cells. The cells were replated on 150-mm TCP at a density of ~ 2500 cells/cm^2^. The process of aggregation and replating was counted as one cycle (or one cyclic-aggregation passage, CP) and the whole process was carried out for 12 cycles (stopped at CP15) to match the passage number of planar culture expansions (P15). For ISR experiments, planar and aggregation cultures were treated with either GSK-2606414 or salubrinal every other passage^31^.

### Real-time reverse transcriptase-polymerase chain reaction (RT-PCR) analysis

hASCs were washed with ice cold PBS and a cell lifter was utilized to remove cells. Cellular suspension was then pelleted at 800 g for 5 min at 4 °C. Samples were then suspended in RNeasy Mini Kit (Qiagen, Valencia, CA) lysis buffer and homogenized with a Sonic Dismembrator 100 (Fisher Scientific, Hampton, NJ) RNA was then fully extracted according to the kit’s instructions. 2 µg of total RNA was used to carry out Reverse transcription, with anchored oligo-dT primers (Operon, Louisville, KY) and Superscript III (Invitrogen, Grand Island, NY). Specific primers for target genes were designed in the software Oligo Explorer 1.2 (https://oligo-explorer.software.informer.com/1.2/. Genelink, Hawthorne, NY). Vinculin was used as an endogenous control for normalization. RT-PCR reactions were performed on an ABI7500 instrument (Applied Biosystems, Foster City, CA), using SYBR Green PCR Master Mix. After amplification, the quality and primer specificity were verified. Variation in gene expression was evaluated using the comparative Ct method: ${2}^{{-(\Delta C}_{t treatment}-\Delta {C}_{t control})}$, based on the expression of the target gene (normalized to *TUBA*).

### Absolute telomere length quantification

Plates containing cells were triple washed with ice-cold PBS before by process according to the Absolute Human Telomere Length Quantification qPCR Assay Kit (ScienCell, Carlsbad, CA). Briefly, purified DNA was acquired using the Quick-DNA/RNA Microprep Plus Kit (Zymo, Irvine, CA) according to the manufacturer’s instructions. Samples were run in either with telomere primer or with the included SCR primer. 2 ng of sample DNA were extracted, and reaction mixture was made by adding 2 μL primer, 10 μL 2× qPCR master mix, and nuclease-free water up to make 20 μL of mix. Samples were sealed and centrifuged at 1500*g* for 15 s. At this point the samples were thermocycled in an ABI7500 instrument without ROX passive reference dye. Telomere length was determined by (*Reference sample telomere lengt*ℎ) ∗ 2 − (Δ*Cq*(*TEL*) − Δ*Cq*(*SCR*)).

### Senescence-associated β-Galactosidase activity

SA-β-galactosidase activity was measured using with the 96-well cellular Senescence Assay Kit (Biolabs, San Diego, CA) according to the manufacturer’s instructions. In brief, cells were seeded at 80% confluence (4500 cells/cm^2^) in CCM and were cultured as normal overnight. Cells were then washed with ice cold PBS, and 1X Cell Lysis Buffer was added, and the cells were incubated at 4 °C for 5 min before lysates were collected and centrifuged for 10 min at 4 °C and 14,000*g*. Supernatant was collected, and a bicinchoninic acid assay was run to determine protein content in each sample. 50 μL of lysate was transferred to a 96-well plate and 50 μL of 2× Assay buffer was added. This was incubated at 37 °C for 2 h, and 50 μL of sample was added to a black fluorescence safe 96-well plate in triplicate. 200 μL of stop solution was added to each sample and the fluorescence intensity was measured (360 nm Excitation/465 nm Emission).

### Western blot assay

The analyzed cells were washed with PBS, and lysed in radio-immunoprecipitation assay (RIPA) buffer (150 mM sodium chloride, 1.0% Trition X-100, 0.5% sodium deoxycholate, 0.1% sodium dodecyl sulfate, 50 mM Tris, pH 8, 2 µg/mL Aprotinin, 5 µg/mL Leupeptin, 5 µg/mL Antipain, 1 mM PMSF protease inhibitor), and homogenized by sonification using a Sonic Dismembrator 100 (Fisher Scientific, Hampton, NJ). Samples were then digested for 20 min on ice, and spun down at 14,000 rpm for 20 min. The supernatant was collected and a Bradford assay was carried out to determine protein concentration. About 20 µg of each sample was denatured at 95 °C in 2× Laemmli Sample buffer. Proteins were separated by 15% BIS–Tris-SDS gels and transferred onto a nitrocellulose membrane (Bio-rad, Hercules, CA). The membranes were blocked for 30 min in 3% skim milk (w/v) in Tris-buffered saline (10 mM Tris–HCl, pH 7.5, and 150 mM NaCl) with 0.1% Tween 20 (v/v) (TBST), or in 3% bovine serum albumin in TBST. Membranes were incubated overnight with the primary antibody diluted in the blocking buffer at 4 °C. Afterward, the membranes were washed four times for 10 min each with TBST and then incubated with an IR secondary (LI-COR, Lincoln, NE) at 1:10,000 for 180 min at room temperature. Blots were washed another four times and processed using the LI-COR Odyssey (LI-COR). Images were analyzed using ImageJ 1.46r software (https://imagej.nih.gov/ij/, National Institutes of Health, USA) and the band density of proteins of interest was normalized to the band density of endogenous control vinculin.

### Adipogenic differentiation

The cells were grown to confluence before CCM was switched for Adipogenic Induction media (AI) containing: high glucose DMEM (Gibco, Grand Island, NY), 10% FBS, 1% penicillin/streptomycin, 0.2 mM indomethacin, 0.5 mM isobutyl-1-methyl xanthine, 1 µM dexamethasone, 10 µg/mL insulin, and 44 mM sodium bicarbonate. The AI media were replaced after two days by Adipogenic Maintenance media (AM) which contains: high glucose DMEM, 10% FBS, 1% penicillin/streptomycin, 10 µg/mL insulin, and 44 mM sodium bicarbonate. After two more days, AM media were replaced by AI media. This cycle was continued for 28 days. At this point, the plates were washed with PBS and fixed with 10% neutral buffered formalin for 1 h at room temp. Plates were washed again before treated with 60% isopropanol for 5 min. The isopropanol solution was then removed, and Oil Red O suspended in 60% isopropanol was added and incubated at room temperature for 5 min. The plate was then washed under tap water until clear and counter stained with hematoxylin for one minute. After washing with water, the plates were imaged under an Olympus IX70 microscope.

### Osteogenic differentiation

The cells were grown to confluence before CCM was switched for osteoblast differentiation media containing: high glucose DMEM (Gibco, Grand Island, NY), 10% FBS, 1% penicillin/streptomycin, 100 mM dexamethasone, 10 mM sodium-β-glycerophosphate, and 0.05 mM ascorbic acid–2–phosphate. The media were changed every three days until day 20. At this point, the plates were washed with PBS and fixed with 10% neutral buffered formalin for 1 h at room temperature. Von Kossa staining was used to identify the matrix mineralization after osteogenic differentiation. The cells were washed before treated with 5% silver nitrate under ultraviolet light for 90 min. After washing three times with deionized water, the plates were treated with 5% sodium thiosulfate for 5 min, and counterstained with Nuclear Fast Red. The cells were then imaged under an Olympus IX70 microscope.

### Colony forming unit (CFU) assay

The analyzed cells were seeded in CCM at 12 cells/cm^2^ and cultured in a 5% CO_2_ incubator at 37 °C for 14 days. The cells were then triple washed with PBS and stained with 0.5% crystal violet (in methanol) for 5 min. The cells were washed with PBS and the numbers of colonies were counted.

### Statistics

A two-tailed Student’s t test was used to compare two groups, and for multiple group comparison a one-way analysis of variance with Tukey’s host-hoc analysis was performed using SPSS software (https://www.spss.com. Chicago, IL, USA). All data are presented as a mean value with its standard deviation indicated (mean ± SD). Differences were considered to be statistically significant when the *p* values were < 0.05.

## Supplementary information

Supplementary Information.
