# Supplementary material for: Cyclical aggregation extends in vitro expansion potential of human mesenchymal stem cells
Source: Sci Rep. 2020 Nov 24;10:20448. doi: 10.1038/s41598-020-77288-4 (PMC7686385; doi:10.1038/s41598-020-77288-4)

**Supplementary Materials**

**Cyclical Aggregation Extends *In Vitro* Expansion Potential of Human Mesenchymal Stem Cells**

Brent M. Bijonowski^1, #, *^, Xuegang Yuan^1,2^, Richard Jeske^1^, Yan Li^1,*^, Samuel C. Grant^1,2^

1. Department of Chemical and Biomedical Engineering, FAMU-FSU College of Engineering, Florida State University, Tallahassee, FL 32310
2. The National High Magnetic Field Laboratory, Florida State University, Tallahassee, Florida.

**Supplementary Figure S1. Example images of human adipose derived stem cell (hASC) aggregates before and after trypsinization.**

**
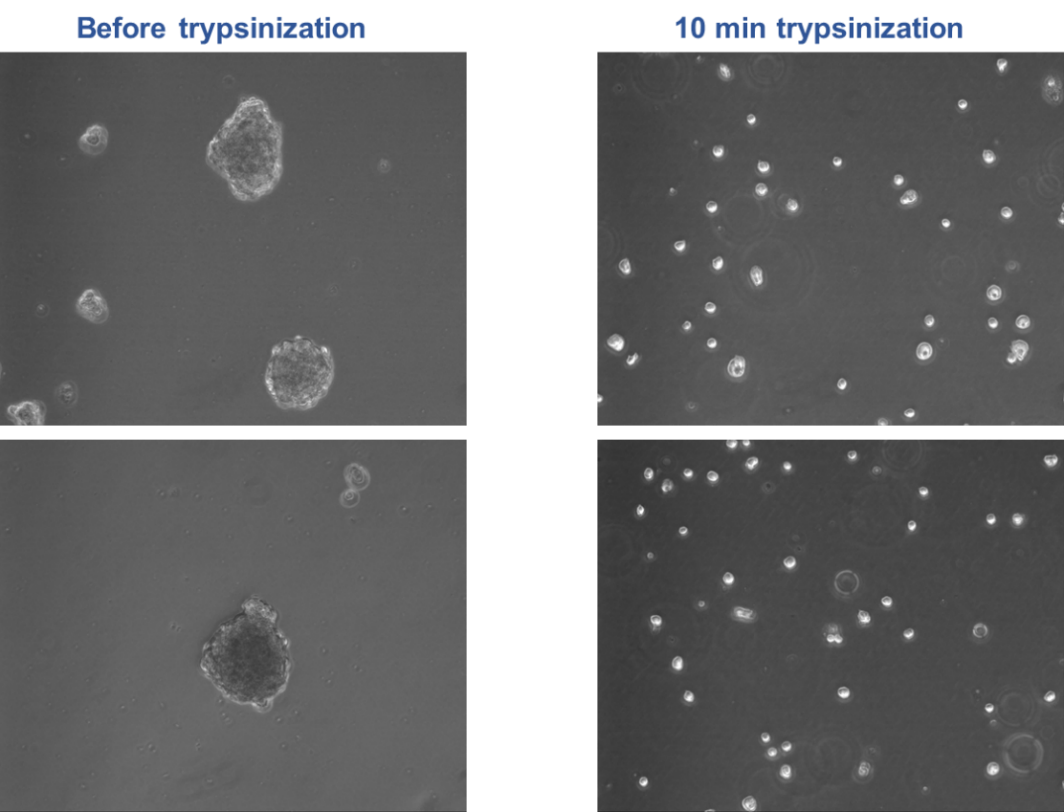
**

**Supplementary Figure S2.** Microscopy images of hASCs after planar culture and aggregate culture with and without treatments by integrated stress response (ISR)-modulation small molecules.


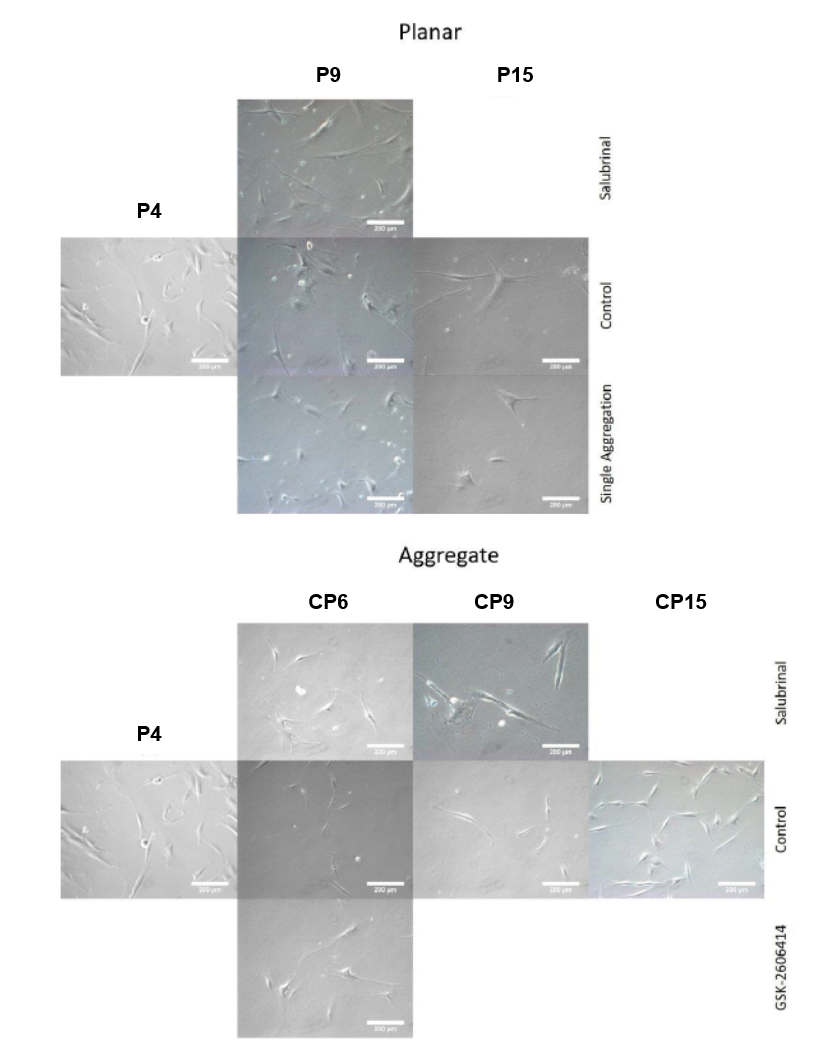


**Supplementary Figure S3. Images of CFU assay results.** Examples show the high CFU numbers and low CFU numbers from hASCs.

**
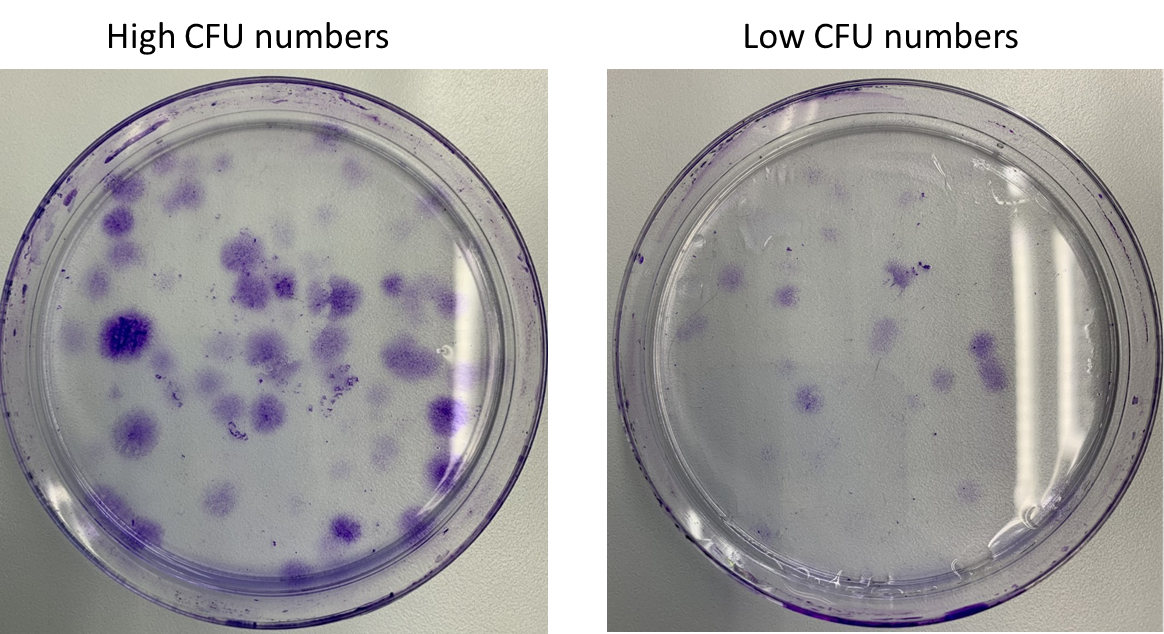
**

**Supplementary Figure S4.** Adipogenic differentiation images from hASCs at different passages with and without aggregation. Oil Red O staining shows the lipid droplets of adipocytes. White scale bar: 400 μm


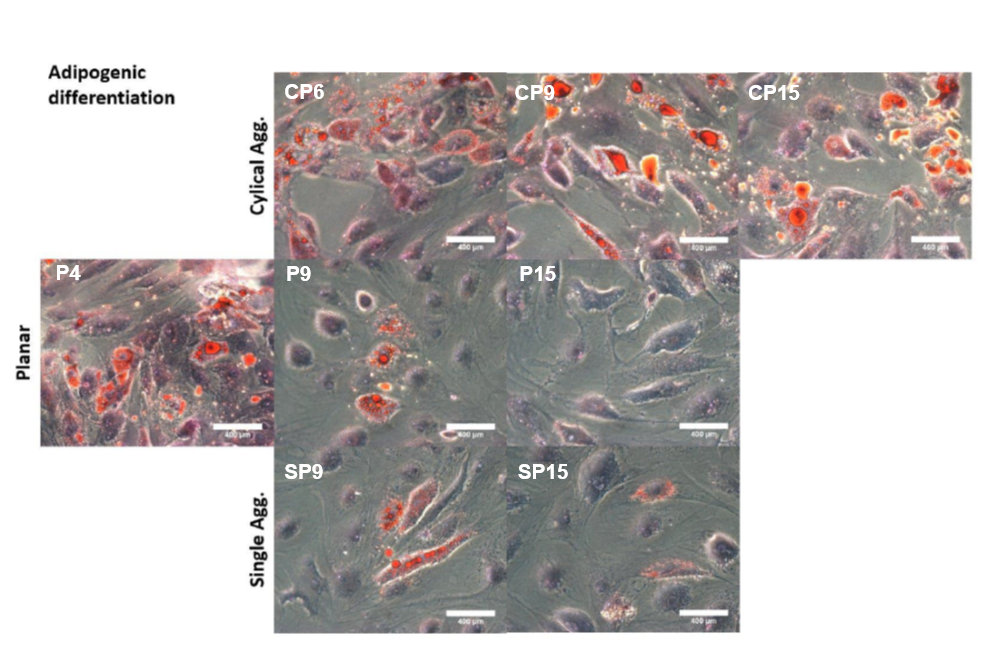


**Supplementary Figure S5.** Osteogenic differentiation images from ASCs at different passages with and without aggregation. Von Kossa staining was used to identify the matrix mineralization after osteogenic differentiation. White scale bar: 400 μm.


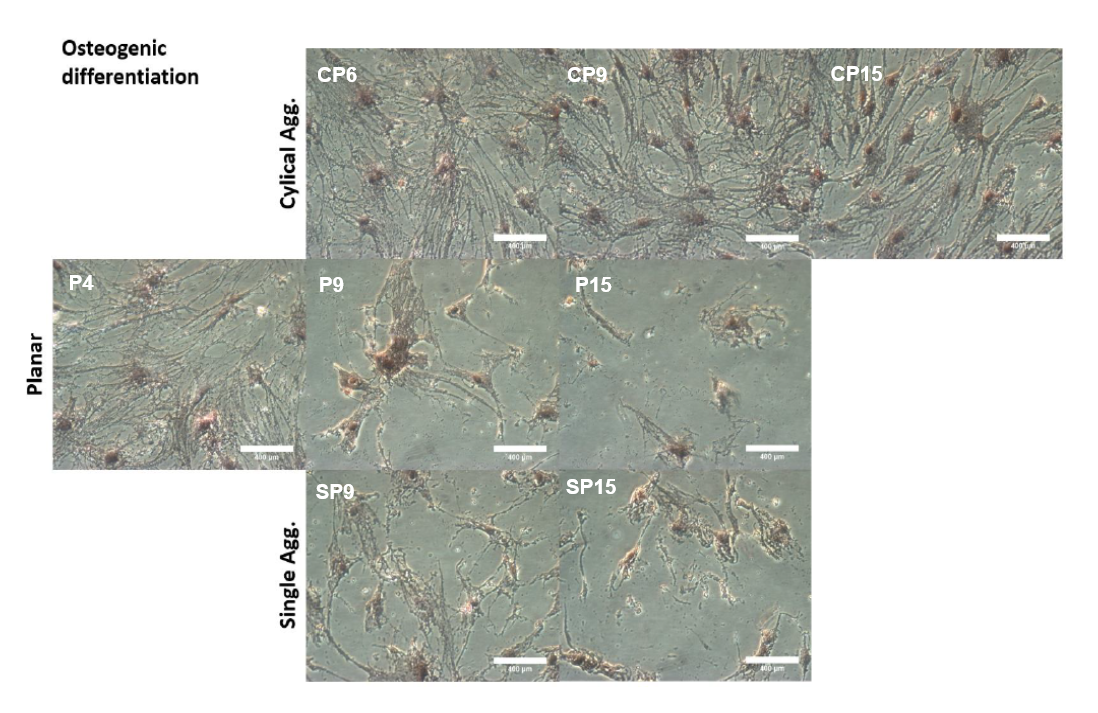

Supplement: Supplementary file 1 — Supplementary Information. [file 41598_2020_77288_MOESM1_ESM.docx]
